# RASMI: Global ranges of building material intensities differentiated by region, structure, and function

**DOI:** 10.1038/s41597-024-03190-7

**Published:** 2024-04-23

**Authors:** Tomer Fishman, Alessio Mastrucci, Yoav Peled, Shoshanna Saxe, Bas van Ruijven

**Affiliations:** 1 Institute of Environmental Sciences (CML), Faculty of Science, Leiden University, 2300 RA Leiden, Netherlands; 2 International Institute for Applied Systems Analysis (IIASA), 2361 Laxenburg, Austria; 3 School of Sustainability, Reichman University, Herzliya, 4610101 Israel; 4 Department of Civil and Mineral Engineering, University of Toronto, Toronto, Ontario M5S 1A4 Canada

**Keywords:** Sustainability, Environmental impact, Environmental social sciences

## Abstract

The construction materials used in buildings have large and growing implications for global material flows and emissions. Material Intensity (MI) is a metric that measures the mass of construction materials per unit of a building’s floor area. MIs are used to model buildings’ materials and assess their resource use and environmental performance, critical to global climate commitments. However, MI data availability and quality are inconsistent, incomparable, and limited, especially for regions in the Global South. To address these challenges, we present the Regional Assessment of buildings’ Material Intensities (RASMI), a new dataset and accompanying method of comprehensive and consistent representative MI value ranges that embody the variability inherent in buildings. RASMI consists of 3072 MI ranges for 8 construction materials in 12 building structure and function types across 32 regions covering the entire world. The dataset is reproducible, traceable, and updatable, using synthetic data when required. It can be used for estimating historical and future material flows and emissions, assessing demolition waste and at-risk stocks, and evaluating urban mining potentials.

## Background & Summary

Globally, humanity is faced by parallel challenges of delivering reasonable quality of life to a growing population while simultaneously reducing total primary resource use, greenhouse gas emissions and other environment impacts. The built environment, made up of buildings and infrastructure, is an important moderator of both. On one hand, buildings and infrastructure stock provide innumerable societal services, including dwellings, transportation, communication, and capital^1^. Buildings and infrastructure further determine the shapes of cities and have major roles in influencing quality of life and the urban environment. Construction materials play core roles as “provision systems” that link human wellbeing with planetary processes^2–4^. On the other, global primary resource use is increasingly straining planetary boundaries. Approximately half of all materials excavated and manufactured globally each year are for construction^5^. By 2050 construction resource use is projected to expected to double the 40 billion tonnes used in 2010 to up to 90 billion tonnes per year^6^. Accordingly, construction materials have received increased attention in sustainability sciences due to their dominance in material consumption and related carbon footprints^7–10^. We know that construction is inducing the extraction and manufacturing of massive quantities of materials but data on where, how, and for what purposes these construction materials are used remains sparse. This limited data and knowledge hampers the policy makers, planners and engineers’ ability to improve on the current status quo of construction, material use and associated environmental degradation. While over the last 5 years big efforts have been made to increase data and knowledge on construction resource use^11–15^, progress remains slow, methodologically heterogenous, and geographically limited. Given the imperatives to act now on material use efficiency all over the world, a faster, more complete method and dataset of global resource use for construction is needed. This paper works towards this gap by gathering and standardizing existing data from around the world, this data is enhanced by synthetic data to provide material use estimates in buildings for the entire globe that are useable now and updatable as bottom up data hopefully continues to improve.

Material intensity (MI) coefficients are a measure of the amount of construction materials embedded in a building, relative to its floor area. MIs are calculated by dividing the total mass of the building materials (e.g. structural, enclosure, and finishes) by the total floor area of the building, leading to a ratio of the average mass of construction material per unit of floor space (kg/m^2^) which is a proxy for the function of the building. This measure provides insights into the material efficiency of a building. Moreover, it is a simple metric for assessing the environmental impacts of a building’s construction materials and life cycle^16^, and is useful for comparing different buildings and their material choices^17^, embodied emissions^18^, and energy designs^19^. The MI values of buildings are affected by a variety of factors^18^, including structural system, building envelope, and interior finishes, as well as by architectural designs, construction technologies and techniques, climate and land conditions, local regulations, and function (e.g. a medical building hosting an MRI machine has different floor slab requirements than a residential building).

Combined with floor space statistics, MIs are used to describe buildings and their material compositions, enabling estimates of the total materials accumulated in historical building stocks and projections of the materials needed to build future buildings. There is a growing body of literature that utilizes MIs in meso- and macro-scale assessments of the built environment, including assessing demolition waste^20,21^, sectoral environmental impacts and emissions such as embodied GHG^22,23^, scenarios of future material flows and their future impacts^24,25^, integrated assessment models (IAMs)^12,26,27^, and spatially explicit building stock accounts to map urban mining potentials^28–31^, socio-economic development trends^32–34^, and at-risk stocks^35–38^.

Material intensity data requires access to detailed construction or demolition records to calculate accurately and hence has previously been time consuming and difficult to obtain. In recent years, however, several datasets of MIs have become available, from individual building by building efforts to larger review datasets based on gathering literature or construction company self-reporting. There are now a few relatively rich country-specific datasets describing dozens to hundreds of MIs of various building archetypes for China^13^, the Netherlands^14^, Canada^15^, Germany^39^, Sweden^40^, and Europe^41^. Furthermore, Heeren and Fishman^11^ and Marinova *et al*.^12^ compiled large sets of MI data gathered from the MI and LCA literature, which focuses mostly on individual case studies. Recent updates to the Heeren and Fishman dataset which incorporates the Marinova compilation and other sources (https://github.com/nheeren/material_intensity_db/) reveal that despite the rapid growth of literature that reports material intensities, several knowledge and methodological gaps remain:

- Even with years of effort by multiple researchers, the available data is measured by the hundreds of datapoints. Given even one city can have hundreds of thousands of buildings, it is unlikely that large data (e.g. 10,000 + data records) will be available in this field within the decade.
- Incomplete and unbalanced geographical coverage. 90% of the 911 datapoints in the updated Heeren and Fishman dataset describe MI values in Western Europe, North America, China, and Japan. Several global regions – notably in the Global South – have no or very few data.
- MI estimation methods and data reporting formats are varied, usually ad-hoc and unique to each study. For example, some studies^28,32,33,42^ distinguish between buildings’ functional use types (e.g. residential, commercial, etc.), others^35,38^ distinguish between structural construction types (e.g. reinforced concrete, timber frame, etc.), or between urban and rural buildings^43–45^, and in some cases differentiate MIs by age classes^30,44^. Very few studies distinguish multiple such features, and many don’t do so at all. Compounding this issue, categorization criteria and even definitions of materials, building functions, and definitions of floor area vary across studies and are only sporadically described in detail. Attempts to create systematic reporting schemes^15,46^ have not yet been adopted by the research community.
- There exists hardly any study of the consistency, representativeness, variance, and uncertainties of MIs, both within individual studies and across sources, despite the clear expectation of variances and uncertainties due to the uniqueness of individual buildings^47^. Individual case studies sometimes compare their MI values to the literature. Yet research specifically focusing on this was pioneered by Schiller *et al*.^48^ who compared MI values and their accompanying attributes between Germany and Japan. The Saxe research group compared residential MI values both within Canada^49^ and between Canada and other countries^50^, also including uncertainty scoring for their data. Moving beyond descriptive statistics, Zhang *et al*.^51^ assessed the variability of MIs in the Chinese database, using a machine learning approach. Nasiri *et al*.^52^ assess the causes of variability of MI values in Finnish wooden houses, as do Miatto *et al*.^53^ innovatively with simulated MI values using building information modeling (BIM). Lederer *et al*. explore the representativeness of MIs^47^. These are, to the best of our knowledge, the only such systematic assessments so far.

Many of these challenges are further described in the comparative MI studies cited above^48–51^ and in recent reviews of material stocks research^7,8^.

Researchers and policy makers that utilize MIs are therefore limited to data describing building types and regions that are well-represented in the body of literature, or are forced to revert to using simple averages and to direct copying of MIs from one region or building type to others. These solutions are generally not representative and do not capture variability and uncertainties, yet the ability to test the applicability or implications of such simplified assumptions is limited due to the lack of data to compare against. The limited research that does exist suggest large variations in material types, architecture and construction norms between countries^50,54^. This hinders the accuracy of both research and policy based on available values, but is hard to correct as the generation of new local MI data is very time consuming. Given the temporal imperatives of resource shortages, the circular economy and GHG emission reductions actions must then proceed without accuracy.

To address some of these gaps, Vilaysouk *et al*.^55^ classified datapoints from Heeren and Fishman’s MI compilation^11^ into clusters with common attributes leading to distributions of cluster-specific MIs, and proposed a decision tree approach to choose MI values from the appropriate cluster. However, that approach was limited by data availability to residential buildings without further differentiation, e.g. by region or construction period. Zhang *et al*.^51^ presented a MI calculator based machine learning analysis on Chinese MI data.

Here, we present the Regional Assessment of buildings’ Material Intensities (RASMI), a new dataset and accompanying estimation method that aims to fill the gaps described above.

The aim of the RASMI dataset is to provide researchers and policy makers with a consistently defined set of MI values for each of 32 regions, comprehensively covering the entire world. By combining publicly available data into a much larger MI database and by using geographic, structural types and function related sampling approaches to develop estimates of MI for any country in the world. The dataset prioritizes the following criteria:

- Comprehensive and exhaustive: a full set of non-parametric ranges (distributions) of MI values for construction materials that are differentiated by region, buildings’ functional use types, and structural construction types, covering the entire world. The RASMI dataset is structured in four dimensions:
  1. 8 materials (concrete, steel, bricks, wood, glass, copper, aluminum, and plastics).
  2. 4 structural construction types (reinforced concrete structure, masonry structure, timber structure, and steel frame structure).
  3. 3 functional use types (Residential single-family, residential multifamily, and non-residential).
  4. 32 global regions compatible with global IAM applications like the Shared Socioeconomic Pathways (SSP)^56^.
  Each datapoint is a range of values that represent one of the unique combinations of these dimensions yielding 8 × 4 × 3 × 32 = 3072 MI ranges, in other words, 384 function-structure-region combinations for each material. For instance, the material intensity of concrete [material] in steel frame structures [structure type] used for multifamily housing [function type] in Japan [region].
- Reasonable and coherent: The method we developed assesses the existing coverage of MI data in the body of literature for each material-function-structure-region combination, and when necessary incrementally increments the data with further MI values based on similarity criteria. This approach produces MI ranges that extend from fully descriptive to fully synthetic data, accompanied by metrics of their representativeness.
- Reproducible, traceable and updatable: the dataset and its estimation methods and code are fully documented and publicly available, enabling to trace back individual MI values to their sources in the literature. Our approach allows the dataset to be iteratively updated by incorporating newly published MI values as they become available, and can be expanded to further construction materials, further building features, and other end-uses beyond buildings such as roads and other infrastructure.

As such, the RASMI dataset is unique in that it aims to answer the question “which MI data are appropriate for my country/region of interest?”. This is a question that collections of extant MI values such as Heeren and Fishman^11^, Marinova *et al*.^12^, Sprecher *et al*.^14^, and Guven *et al*.^15^ cannot answer. These different datasets fulfil different needs of construction material researchers. It will be useful going forward for the quantity and diversity of such MI data to improve, both improving the availability of ground truth data and estimates produced by the RASMI method. In this Data Descriptor, we present the method and the first public version of the RASMI dataset.

## Methods

The method to create the RASMI dataset consists of 4 steps to create unique MI value ranges for each of the 3072 material-function-structure-region combinations:

1. Collection and labeling the raw MI data collected from the literature with harmonized descriptive definitions of functional use types, structural construction types, regional location, and other dimensionalities or features.
2. Incremental expansion of MI coverage when required, using similarity criteria to create pools of MI values for each material-function-structure-region combination.
3. Identification of materials with poor MI coverage for which unique material-function-structure-region combinations cannot be differentiated.
4. Calculation of non-parametric statistics for each combination for inclusion in the dataset.

These steps are detailed below.

### Labeling of the raw MI data

The source for raw MI data is the updated version of the Heeren and Fishman MI database^11^, here referred to as H&F for short. The H&F MI database consolidates MI data found in the literature to create a meta dataset.

Individual datapoints in the H&F database cover between one and 21 construction materials out of 32 possible materials. The H&F database describes each datapoint as-is, maintaining the original description and definition from the source publications to preserve accurate descriptions of the data. This results in varying definitions and descriptions of buildings, reflecting the ad-hoc data creation processes in the data sources, but also reflecting the variability of buildings.

We added to the raw H&F database five new features (i.e. columns) that label the data in a harmonized fashion: structural construction type, functional use type, region, energy efficiency, and data quantification method. We labeled each of the H&F datapoints by assigning them one attribute for each feature from the lists in Table 1.

**Table 1:** Harmonized features and their possible labels used to label the datapoints in the Heeren and Fishman (H&F) MI database.

Feature	Structural construction types [14 in 5 groups]	Functional use types [17 in 6 groups]	Region [32 in 5 groups]	Energy efficiency [4]	Quantification method [3]
**Possible labels**	**S.1 Reinforced concrete structure**	**F.1 Residential single-family**	**R.1 OECD and EU member states and candidates**	**E.1 Standard**	**Q.1 Real-world single case study**
	S.1.1 With concrete floor	F.1.1 Detached	R.1.1 Australia and New Zealand	**E.2 Efficient**	**Q.2 Sample/average/statistics**
	S.1.2 Prefabricated	F.1.2 Row house	R.1.2 Canada	**E.3 Zero energy**	**Q.3 Modeled/hypothetical/regulations**
	S.1.3 Unspecified	F.1.3 Unspecified	R.1.3 Eastern Europe (excl. former Soviet Union and EU members)	**E.4 Unspecified energy**	
	**S.2 Steel frame structure**	**F.2 Residential multifamily**	R.1.4 EFTA (Iceland, Norway, Switzerland)		
	S.2.1 With concrete floor	F.2.1 Low	R.1.5 European Union member states that joined prior to 2004 (EU-15)		
	S.2.2 With steel floor	F.2.2 High	R.1.6 EU member states that joined as of 2004 - high income		
	S.2.3 Unspecified	F.2.3 Tower	R.1.7 EU member states that joined as of 2004 - medium income		
	**S.3 Timber structure**	F.2.4 Unspecified	R.1.8 Japan		
	S.3.1 Traditional wood	**F.3 Residential unspecified**	R.1.9 Republic of Korea		
	S.3.2 Engineered wood	**F.4 Non-residential**	R.1.10 Turkey		
	S.3.3 Unspecified	F.4.1 Offices, low	R.1.11 United States of America		
	**S.4 Masonry structure**	F.4.2 Offices, high	**R.2 Reforming Economies of Eastern Europe and the Former Soviet Union**		
	S.4.1 Bricks	F.4.3 Retail	R.2.1 Central Asia		
	S.4.2 Stone	F.4.4 Factory	R.2.2 Eastern Europe, former Soviet Union (excl. Russia and EU)		
	S.4.3 Adobe or mud	F.4.5 Warehouse	R.2.3 Russian Federation		
	S.4.4 Unspecified	F.4.6 Civic	**R.3 Asian countries excl. the Middle East, Japan and Former Soviet Union**		
	**S.5 Unspecified structure**	F.4.7 Unspecified	R.3.1 China (Mainland, Hongkong, Macao)		
		**F.5 Informal**	R.3.2 Indonesia		
		**F.6 Unspecified function**	R.3.3 India		
			R.3.4 former Centrally Planned Asia		
			R.3.5 Other Asia - low income		
			R.3.6 Other Asia - medium and high income		
			R.3.7 Pakistan and Afghanistan		
			R.3.8 Taiwan		
			**R.4 The Middle East and Africa**		
			R.4.1 Middle East Asia - high income		
			R.4.2 Middle East Asia - low and medium income		
			R.4.3 North Africa		
			R.4.4 South Africa		
			R.4.5 Sub Saharan Africa - low income		
			R.4.6 Sub Saharan Africa - medium and high income		
			**R.5 Latin America and the Caribbean**		
			R.5.1 Brazil		
			R.5.2 Latin America - low income		
			R.5.3 Latin America - medium and high income		
			R.5.4 Mexico		

We reviewed the datapoints’ sources for relevant descriptive information for these labels and when possible, contacted the original authors for further data. Structure type [S], function type [F], and energy efficiency [E] and their subtypes include “Unspecified” labels, for cases in which the sources’ descriptions are general, unknown, unclear, represent multiple types equally, or represent types other than the listed ones. The structural construction type [S] labels reflect the load bearing structure. For example, a building described in its source publication as “conventional structure in reinforced concrete, masonry in ceramic blocks”^57^ is labeled as one of the subtypes of reinforced concrete structure [S.1], not as masonry structure [S.4]. The functional use type [F] labels reflect the major function described in the data source. The thresholds between the residential multi-family [F.2] subtypes are low [F.2.1] up to 5 floors, high [F.2.2] between 7 and 19 floors, and tower [F.2.3] over 20 floors, or as defined in the source publication. The civic non-residential subtype [F.4.6] includes schools, hospitals, utilities, municipal buildings, libraries, etc.; and the informal type [F.5] describes informal construction such as shacks and shanties. The definitions of the regions and subregions [R] are taken from the SSP database^56^. The energy efficiency [E] and the quantification method [Q] labels follow the source publications’ own explicit or implicit descriptions. To limit ‘combinatorial explosion’ and due to the limited variance of energy efficiency [E] and quantification method [Q] in the data, we use only the top-level labels of structure type [S] and function type [F] except for the informal functional use type [F.5] in the next steps.

The most recent update of the H&F database incorporates several other MI datasets from recent years including Marinova *et al*.^12^, Sprecher *et al*.^14^, Guven *et al*.^15^, among others. The additional data were identified and harmonized with the H&F database using the methods described in its data descriptor article^11^, and the details of updates and newly added sources are tracked on its GitHub repository (https://github.com/nheeren/material_intensity_db). Since its original publication in 2019^11^, it has tripled in size from 301 to 910 individual MI datapoints, including 332 points newly added for this project, and it now covers 51 countries and regions, collated from 115 scientific publications (Table 2).

**Table 2:** Statistics of the coverage of data by material, used to determine the approach to estimate ranges of MI values for each material-function-structure-region combination.

Material	Number of raw MI datapoints (out of 906)	Coverage of function-structure-region combinations (out of 384 possible combinations per material)	Gini coefficient	MI data sources
Concrete	812	91%	0.685	^14–17,19–24,28–33,35,36,38–42,44,45,47,57,61–134^
Wood	759	85%	0.725	^14–17,19–24,28–33,35,36,38–42,44,45,61–117,135–141^
Steel	698	79%	0.706	^14–17,22–24,28–33,35,36,38–45,61–63,65–70,72–79,81,83,84,86–91,93,95,98,99,101–103,105–113,118–130,136–140,142–145^
Glass	498	69%	0.746	^14–17,19,21,23,24,28–32,35,39,40,44,45,61–63,65–71,75–78,80–88,90,91,93,94,96,98,99,101–104,118,120–124,141,142,146,147^
Brick	464	54%	0.811*	^14,15,20,21,23,24,28,29,31–33,39–42,44,45,61–63,70,71,74,75,81,84–100,119,121,122,132,137,138,145^
Plastics*	186	33%*	0.888*	^15,21,28,31,39,40,61,62,65,67,71,80–88,118–120,133,138^
Aluminum*	153	36%*	0.844*	^15–17,19,28,29,31,35,39,61–79,118,119,131,138,142,147^
Copper*	81*	12%*	0.953*	^28,40,61–63,118,138,148^

*indicates a statistic that doesn’t pass the criteria.

These source data most consistently contained information on the structural materials use in buildings, with decreased granularity and availability of data on architectural finishes and/or mechanical, electrical and plumbing. Accordingly, main structural materials are the focus of our assessment.

**Fig. 1:**
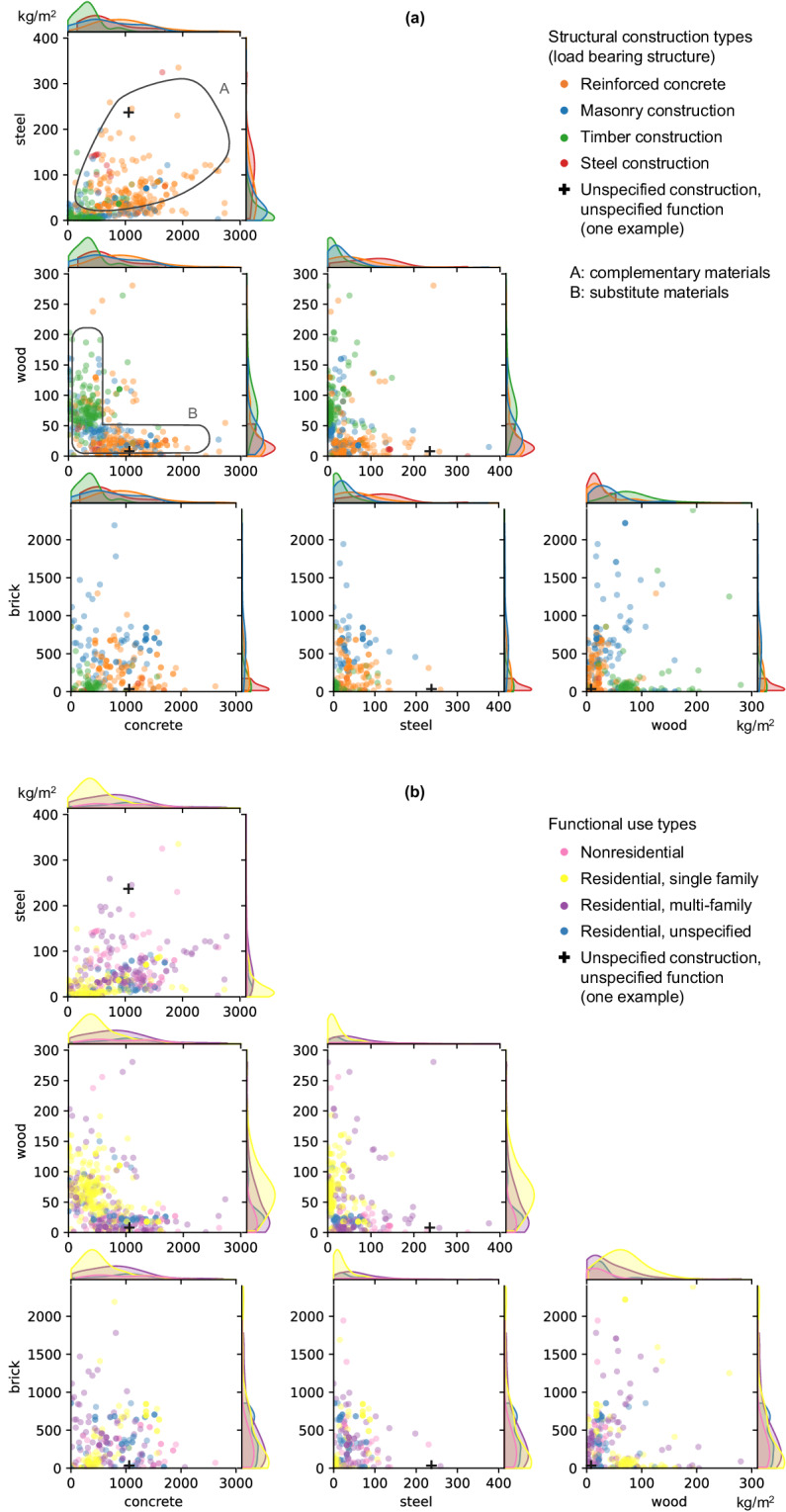
Pairwise (two at a time) scatterplots and distributions of concrete, steel, wood, and bricks material intensities (kg/m^2^) of the Heeren and Fishman database^11^, classified by (**a**) buildings’ structural structure types, and (**b**) functional use types. Refer to the main text for the annotations in panel (**a**).

The H&F data supports observations of previous studies^50–53,55^ that MI values correlate most to construction structural types, corresponding the [S] label of our data, and less so to function types [F]. Figure 1 visualizes this with two versions of the same set of pairwise MI scatterplots, colored by structural type in panel a or function type in panel b. Each subplot visualizes a pair of the four structural materials (concrete, bricks, wood, and steel) and every scatterpoint is a datapoint in the H&F database. The distributions of each material per structure type are added on the edges of the scatterplots.

Figure 1a shows that structural construction types [S] tend to have relatively unique MI value ranges, which manifest as identifiable areas of same-colored areas across the pairwise plots. For example, the space occupied by scatterpoints in the concrete-steel panel shows that in these two materials are complementary (annotated A in Fig. 1a): generally, more concrete means more steel. This complementarity is especially prominent in reinforced concrete structures. In comparison, the general L-shape of the distribution of points in the concrete-wood scatterplot implies that these two materials act as substitutes in construction (annotated B in Fig. 1a): there is either relatively high wood MI with low concrete MIs, mostly in timber structures, or low MIs of wood but high MIs of concrete in reinforced concrete structures, and these two structure types occupy different spaces in the plot. This is in contrast to the visualization of the same data but colored by functional use types in Fig. 1b. Only the single-family residential data occupy fairly unique spaces in the pairwise panels of Fig. 1b, and the distributions of MIs of the other function types rather overlap in space. These observations are supported by nonparametric statistical tests (Kruskal-Wallis, Kolmogorov-Smirnov, and Anderson-Darling tests, code and results available in the repository).

These observations suggest that knowing the MI values of the four structural materials can infer the structural construction type [S] of a building, but not the functional use type [F]. This is visualized with a single datapoint marked by a + in Fig. 1, for which both the structure and function were not specified in the data source. Based on its location in relation to other datapoints in the panels of Fig. 1a, it can be inferred that this datapoint describes a reinforced concrete structure. However, a similar inference cannot be made for its function, which may be nonresidential or multifamily residential.

We thus used supervised machine learning to assign one of the defined structure types for the 237 datapoints whose structure type was labeled as unspecified structure [S.5]. We trained a random forest classifier model using the MI values of the four structural materials (concrete, steel, wood, and bricks) of the 673 datapoints already labeled with a top-level structure type [S.1 – S.4]. We then used the trained model to predict the structure type of the 237 unspecified datapoints. The random forest classifier consistently performed better than other models (neural network, k-nearest neighbors, and logistic regression) in cross validation and random sampling tests of training and test sets. This step is implemented in Python using the Orange suite^58^, and the workflow and test results are available in the RASMI GitHub repository (https://github.com/TomerFishman/MaterialIntensityEstimator).

### Incremental pooling of MI datapoints using similarity criteria

In order to estimate plausible MI ranges for each region and building type, each of the 384 possible function-structure-region combinations per material require a minimum pool of 30 MI value datapoints (we discuss the choice of 30 in the technical validation section). For combinations that do not have 30 datapoints in the database, we iteratively increment the number of datapoints by adding similar datapoints in the following order:

1. MI datapoints of the same structure type [S] and region [R.x.x] but different function type [F] are added: unspecified [F.6] are added to non-residential [F.4], residential unspecified [F.3] are added to the specified residential types [R.1] and [R.2], followed by [F.6].
2. MI datapoints of the same structure type [S] and function type [F] but from the top-level macro-region [R.x] are added.
3. MI datapoints of the same structure type [S] and top-level macro-region [R.x] but different function type [F] are added, with the same rules as in step 1.
4. MI datapoints of the same structure type [S] and function type [F] from all regions [R] are added.
5. MI datapoints of the same structure type [S] and all regions [R] but different function type [F] are added, with the same rules as in step 1.

At every incrementation iteration, the datapoints that are already included are duplicated to give them more weight, so that local data and more closely-similar is represented at higher weight in the estimation of range statistics in the next step. This incremental expansion process halts once at least 30 datapoints (including those duplicated) have been collected, or once the five increment steps have been exhausted.

**Fig. 2:**
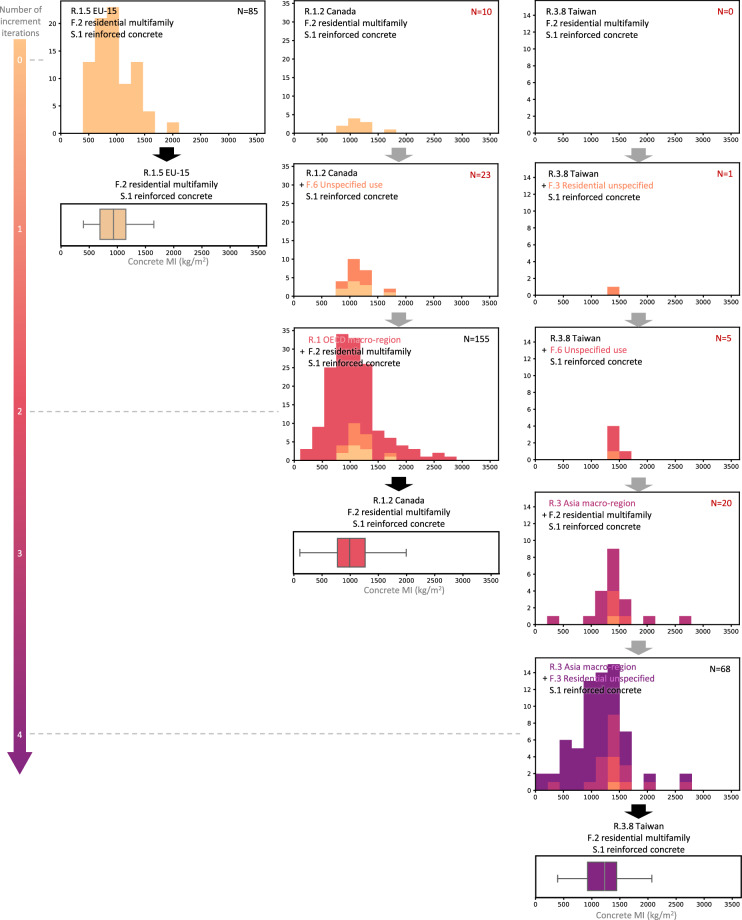
Three examples (of 0, 2, and 4 iterations of increment) of the creation of a pool of datapoints for estimation of concrete material intensity (MI) ranges. The pools are visualized as histograms and the resulting MI ranges as boxplots.

The incremental pooling process is exemplified in Fig. 2. On its left, the extant raw datapoints that describe concrete MIs of residential multifamily buildings with a reinforced concrete structure in the EU15 region are already sufficient (over 30 datapoints) and used as-is to create MI ranges with zero iterations of incrementation. The center is an example of two increment iterations: the number of extant raw datapoints that describe concrete MIs of residential multifamily buildings with a reinforced concrete structure in the Canada region are insufficient. The pool of MIs undergoes two incrementation steps before MI ranges can be created. On the right, no extant datapoints describe concrete MIs of residential multifamily buildings with a reinforced concrete structure in the Taiwan region. The pool of MIs undergoes four incrementation steps before MI ranges can be created. The number of iterations is stored as an indicator of representativeness of the MI data in the datapoints pool for each material-function-structure-region combination. This and the following steps are carried out in Python code, available in the RASMI GitHub repository.

### MI values pooling for materials with poor data

Pooling MI values with the similarity criteria process described above requires diversity and good coverage of MI values to culminate in unique MI datapoint pools for each of the function-structure-region combinations. However, some materials are insufficiently covered in the extant data to create unique MI value ranges for each of the function-structure-region combinations, because the number of datapoints that describe these materials’ intensities is low or because they cover few types of material-function-structure-region combinations.

We identify materials with poor coverage in the extant data if they match at least two of three criteria: (1) less than 15% of the extant raw datapoints in the H&F data^11^ include values for the material in question, (2) these extant datapoints cover fewer than 50% of the 384 possible function-structure-region combinations, and (3) the extant datapoints are unequally distributed among the combinations, i.e. they belong to only a handful of function-structure-region combinations rather than a diversity of combinations, as indicated by a Gini coefficient higher than 0.8. This also highlights areas where future data collection can most contribute to data diversity and coverage.

Table 2 shows that in the extant H&F data, concrete, wood, steel, glass, and brick have good coverage and therefore utilize the similarity criteria approach to increment their MI pools. Copper fails all three coverage criteria, and plastics and aluminum fail two of the three.

For the materials with poor MI data, all of these materials’ raw datapoints are used to create global value ranges for all function-structure-region combinations regardless of function type, structure type, or region. For example, all 186 plastics MI datapoints are used as the same pool for all combinations. Datapoints are given more weight in the pools of the function-structure-region combinations that they originate from. For instance, 47 MI datapoints describe plastics in Canadian [R.1.2] single-family [F.1] timber housing [S.3], so these 47 datapoints get a bigger weight than the other 139 plastics MIs in the Canada-residential single family-timber combination’s pool.

### Calculation of non-parametric statistics

At the end of the previous steps, each of the 3072 material-function-structure-region combinations have pools of at least 30 MI datapoints. We then estimate non-parametric frequency statistics, namely the 0th, 5, 25, 50 (median), 75, 95, and 100th percentiles from these pools. We do not fit any types of probability distribution functions to these pools of datapoints for several reasons. First, there is no theoretical or empirical support in the literature for the type of function that would a-priori describe distributions MI values that we are aware of. Second, as exemplified in Fig. 2, visual inspection of the datapoint pools don’t point toward a generalizable distribution: many pools are asymmetrical, and some are bi- or multimodal. Third, the synthetic MI value pools aren’t a statistical sample from a population of MI values, meaning that no inference can be made on the characteristics of the “true” population. Rather, the non-parametric approach doesn’t assume a distribution, and the percentile statistics form a seven-number summary of value ranges for each of these combinations that match the aim and criteria of our dataset: comprehensive and exhaustive, reasonable and coherent, and reproducible and traceable.

## Data Records

The RASMI data record is available in Zenodo^59^, mirroring versions in the RASMI GitHub repository. The file name contains the date of estimation, serving as version number. The Zenodo version used for this data descriptor is v20230905^59^. The MI ranges data record described in this data descriptor is *MI_ranges_20230905.xlsx*.

The data structure has four dimensions: 8 materials × 4 structure construction types × 3 functional use types × 32 regions, leading to 3072 unique combinations. Each combination has a range of MI values representing the unique material-function-structure-region combination. This range is composed of seven percentiles: the 0th (minimum), 5th, 25th (1st quartile), 50th (median), 75th (3rd quartile), 95th, and 100th (maximum) percentiles. For example, the pair of 25th and 75th percentiles form the interquartile range of plausible MIs for a given function-structure-region combination.

The data record of the MI ranges is stored as a Microsoft Excel spreadsheet for ease of use. The spreadsheet contains eight sheets, one for each material. The sheets are identically structured. The columns describe the function-structure-region combination, the SSP macro-region, the number of MI datapoints originally in the H&F database, the number of increments to add further MI datapoints, the final number of MI datapoints after incrementation, and the seven percentile values described above. The rows are the 384 function-structure-region combinations for that material.

Table 3 demonstrates the data record structure, with the first two and last two rows of the concrete MI ranges. Row 0 is the concrete MI range for nonresidential reinforced concrete buildings in China, an example of a range estimated as-is from 35 datapoints originally describing such buildings. Row 1 is an example of completely synthetic data, the concrete MI range for nonresidential reinforced concrete buildings in Indonesia, for which no data exists in the H&F database and the MI range is estimated from 38 datapoints collated through one incrementation iteration. The 0th and 100th percentiles values of the two match, reflecting the increment of datapoints from the Chinese ones to the Indonesian ones. Likewise, the MI ranges in row 382 for single-family residential timber structures in the former Soviet Union Eastern Europe region and row 383 for Russia are identical to each other and are composed of 133 MIs pooled together.

**Table 3:** Sample of the data record with the first two and last two for concrete MI ranges.

	function	structure	region5_32	region5	raw_H-F_db_count	increment_iterations	incremented_count	p_0	p_5	p_25	p_50	p_75	p_95	p_100
0	NR	C	ASIA_CHN	ASIA	35	0	35	140.3	420.4	713	1088.7	1568	2195.7	2694.1
1	NR	C	ASIA_IDN	ASIA	0	1	38	140.3	444.7	725.6	1091.8	1625.8	2104	2694.1
⋮														
382	RS	T	REF_EEU-FSU	REF	0	1	133	5	48.5	229.2	351.2	468	861.8	1563.2
383	RS	T	REF_RUS	REF	0	1	133	5	48.5	229.2	351.2	468	861.8	1563.2

Accompanying the main MI ranges data record file is a second Excel file, *MI_data_20230905.xlsx* which contains the raw MI datapoints used to estimate the MI ranges for each material-function-structure-region. For each such datapoint, four attributes are described: its original material-function-structure-region combination, record ID number in the H&F database, value, and in which increment iteration they were added. This accompanying file serves to document the MI pool, to trace back the incrementation process, and enable estimation of other statistics as needed.

## Technical Validation

One of the factors that could potentially influence the MI ranges is the number of raw MI datapoints in the H&F dataset and their values. We tested the influence of this by leaving a random 10% of the 906 H&F datapoints out and re-running the range estimation process with n = 815 MIs. Independently repeating this 20 times, this test provides cross validation results. On average, 49% of the left-out datapoints’ values are within the newly calculated interquartile range (25th–75th percentiles range) of their respective function-structure-region MI range. This percentage grows to 84% datapoints fitting inside the 5%–95% percentile range.

This exercise also enables to test the percent changes in the resulting MI ranges compared to those created with the full data. The relative differences per material and range percentile are presented in Table 4, averaged for all combinations and across the 20 independent runs of this test. Overall, the MI ranges are prone to changes in values subject to the available raw MI data – which matches the objective of this dataset to reflect the availability of MI data. However, in most cases the changes are low. The three central 25th, 50th, and 75th percentiles are relatively insensitive to the reduction of raw MI datapoints, and the edge percentiles are more sensitive. The lowest 0th and 5th percentiles are most sensitive to the availability of data, because they are subject to the values of outlier MI datapoints. Nevertheless, in absolute terms the changes in kg/m^2^ for these two low-end ranges are negligible.

**Table 4:** Percent changes in the values of the MI range percentiles when leaving 10% of the input MI data out.

Percentile	0th	5th	25th	50th	75th	95th	100th
concrete	+1.0%	+0.8%	+1.3%	−0.3%	+0.7%	−0.6%	−1.4%
brick	+11%	+45%	+4.7%	+0.1%	+0.0%	−0.1%	−1.2%
wood	+36%	−0.2%	+1.7%	+1.5%	+0.4%	+0.9%	+2.9%
steel	+25%	+11%	+2.4%	+4.4%	+0.9%	+1.9%	−1.9%
glass	+41%	+7.3%	+2.0%	+0.2%	+2.3%	−1.0%	−2.7%
plastics	+0.0%	−0.3%	−0.2%	+0.1%	−0.7%	+6.2%	−5.3%
aluminum	+0.0%	−0.1%	+8.9%	+2.0%	+7.2%	−7.6%	−1.5%
copper	+1.7%	+49.2%	−0.4%	+1.0%	+2.8%	+10%	−5.0%

The second factor that may influence the MI data ranges is the minimal number of pooled MIs after which the incrementation halts. This number is set at 30 MIs, which serves as a balance between data availability and parsimony. With current raw MI data availability of the H&F dataset, only 22 of the 3072 material-function-structure-region combinations have at least 30 datapoints that can be used directly to draw percentiles of value ranges. Increasing the minimum datapoints to 35 decreases the number of those direct-to-use combinations to 16 or down to 11 if 40 is set as the halting value. This would mean that fewer combinations would be described as-is rather than incremented by MIs from other building types or regions, leading to less representativeness in the results. Additionally, increasing the minimum number of datapoints leads to incrementation with MIs from less-similar regions, leading to the same outcome. Nevertheless, we compared the MI ranges obtained with at least 30 datapoints to the MI ranges that would be obtained if it was set to different values, and the differences are negligible. For example, the average value of the medians change by only 0.4% to 2% if the minimum was set to 10 less or 10 more incremented datapoints, respectively.

## Usage Notes

### Using material intensity ranges

The MI data in RASMI are intended to be used as ranges, to promote the inclusion and communication of the uncertainties inherent in buildings’ materials. For example, using the equation *material stock* = *floor area* × *material intensity*, one can estimate the plausible range of material stocks that compose (and were required to construct) a single-family house with a reinforced concrete structure of 120 m^2^ total floor area in Brazil (Table 5).

**Table 5:** Example of the usage of the material intensity ranges (25th, 50th, and 75th percentiles) to estimate the material stocks of a 120 m^2^ house.

Material	RASMI [R.5.1] Brazil, [F.1] Single-family residential, [S.1] Reinforced concrete MIs (kg/m^2^)			House floor area (m^2^)	Material Stocks (kg)		
	25th	Median (50th)	75th		25th	Median (50th)	75th
Concrete	480.6	807.6	1,114.5	× 120 m^2^ =	57,672	96,907	133,741
Steel	15.6	21.2	35.3		1,872	2,544	4,232
Brick	52.9	234.0	488.9		6,347	28,080	58,667
Wood	19.5	69.0	90.0		2,341	8,280	10,800
Glass	1.51	2.00	2.54		181.4	240.0	304.8
Plastics	0.39	1.20	3.00		47.1	143.9	359.6
Aluminum	0.13	0.49	1.07		15.5	58.8	128.4
Copper	0.08	0.18	0.27		10.1	21.9	32.0

**Fig. 3:**
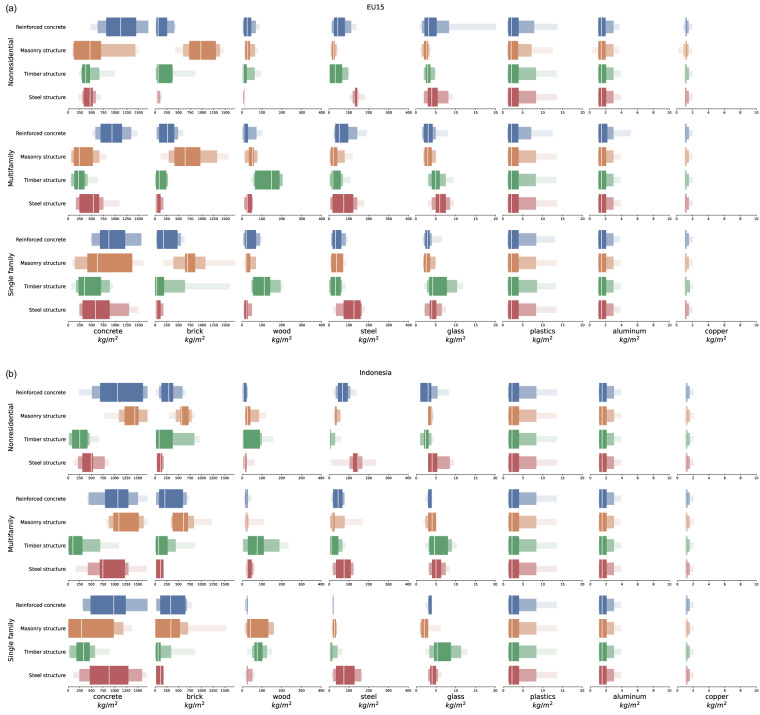
Letter value plot examples of the MI value ranges of the 8 materials (horizontal, note the varying scales), grouped vertically by functional use types and by structural construction type. (**a**) ranges for the EU15 region, (**b**) ranges for the Indonesia region.

The RASMI MI ranges can be visualized as box-letter plots (Fig. 3). In each material-function-structure-region combination, the median (50th percentile, in white in the figure) is the ‘average’ central value of material intensity in units of kg/m^2^ of floor area. The interquartile range (25th and 75th percentiles) in the central thicker boxes in the figure bound the middle half of the MIs pooled for creation of the MI range and so they represent the most plausible ranges of MIs in each combination. These three percentiles fit most uses and applications of MI data.

The 5th and 95th percentiles expand the range to include less-likely MI values, as they describe 90% of the MI pool. The 0th and 100th percentiles are the minimum and maximum MI values in the material-function-structure-region combination’s range, which should be used with attention, because they may originate from unrepresentative outlier data from the H&F database.

Figure 3a exemplifies the MI ranges for the EU15 region. Patterns such as high MI ranges of wood in timber construction and high MI ranges of brick in masonry construction are visible.

The ranges for concrete, brick, wood, steel, and glass are mostly from datapoints originating in the literature (i.e. no iterative increments were required). This can be compared to Fig. 3b that shows ranges for the Middle East Medium Income region, whose ranges are mostly synthetically produced by addition of MI datapoints from similar function types and other regions. The nearly identical ranges of the low-value plastics, aluminum, and copper within and between the two regions reflect the poor representation of these materials in the raw data, which led to the use of all extant datapoints to create their ranges.

The estimated ranges make it clear that material intensities vary greatly both within and between regions, types of structure, and types of building functions. The ranges capture both the aleatory uncertainty (variability) of material intensities between buildings of the same type, and the epistemic uncertainty arising from the different methods, system boundaries, definitions, etc. of the diverse data sources. At this point in time a differentiation between these two uncertainty types^60^ can’t be made for building MI values, and therefore epistemic uncertainties can’t be minimized because of the ad-hoc and arbitrary nature of the data sources. The high top-range MI of glass in nonresidential reinforced concrete in the EU15 in the top row of Fig. 3a exemplifies this. The value of around 20 kg/m^2^ in the extreme end of the range stands out, and it could originate from a measurement error in the source data or from an MI of a true glass-intensive building such as a glass-cladded office tower.

Therefore, the MI ranges of RASMI candidly communicate that both variability and biases exist in MI values. These observations lead us to discourage usage of only the median values as sole point estimates of MIs in studies, and rather to accompany the medians with at least the interquartile range bounded by the 25th and 75th percentiles in order to truthfully convey the embodied uncertainties in building materials research. At the same time, since the H&F database retains outlying values that may end up as the minimum (0th percentile) and maximum values (100th percentile) of the RASMI ranges, we advise using the 5th and 95th percentiles as the extreme MI ranges rather than the 0th and 100th percentiles when describing less common MI values.

The MI ranges data here is reflective of past construction and current norms. It is most useful for estimating the material stock at a neighborhood, city, region or country scale and for estimating future flows of materials to increase building stock. At the scale of an individual building the MIs calculated here will have high uncertainty but can still provide a rough estimate of the materials needed for construction and/or the materials stocked in a building and available for recycling at the end of the life. The foundational data from RASMI comes from existing studies and buildings, as such it represents the range of current practice, uncertainty is higher in translating RASMI to future constructions that deviate in large ways from current construction approaches (e.g. showcase sustainability buildings).

### Material intensity combinations

Certain material MI combinations are unlikely, such as a building with high MIs of both concrete and bricks (which act as substitutes to some extent, compared to the complementarity of concrete and steel), and this is reflected in the differentiation between structure types. Nevertheless, the estimation of the MI ranges is done per material, and so doesn’t address the relative intensity of one material compared to the others within each function-structure-region combination. For example, within the MI ranges of the eight materials in a certain function-structure-region, the RASMI approach on its own cannot prescribe whether it’s more “realistic” to couple the 75th percentile MI value of concrete with the 75th percentile MI value of bricks or to couple it with the 25th percentile of bricks, because this would require understanding of building engineering, which our statistical approach doesn’t include. In practice, one option is to consistently use the same percentile values of all materials to e.g. represent the “heaviest” and “lightest” likely buildings in a function-structure-region combination, as sensitivity scenarios. Furthermore, the raw MI datapoints in the H&F database can be used for a separate analysis of the relative intensities of materials in building types – a characteristic that we implicitly used to label unspecified structure types – though this is outside the scope of the RASMI dataset.

### Functions, structures, regions, and further dimensions

Beyond function types, structure types, and regions, building material intensity values can be differentiated by further dimensions or features such as by energy efficiency or by construction period. Although information for both of these features is included in the raw MI data of the H&F database, we opted not to differentiate combinations by any further features because of the multiplicative nature of such differentiations. For example, adding a differentiation of just two construction periods (e.g. pre- and post-1950) to the current 3072 combinations would double the combinations to 6144 and dramatically reduce the number of extant MIs in each combination. Most of the combinations are already virtually empty prior to incrementing. Adding more dimensions would only intensify this, and make the incrementing process unwieldy and less meaningful.

Similarly, we decided not to differentiate the building material intensities by definition of floor area, because of lack of evidence of MIs differing across floor area definitions and only partial data availability. While this attribute is recorded as part of the H&F database, less than half of the datapoints (49%) can be associated with a clear definition of floor area. Among them, the majority (46% of all datapoints) use gross floor area and just 27 datapoints (3% of all datapoints) use net floor area. Yet, their Mis are within range of the other datapoints and no significant differences could be detected.

### Data updates

The RASMI data will be kept up-to-date by collating MI values into the H&F database as they are found in the literature appear in new publications. The value range estimation process will be re-run with any new MI values at least once a year and the new ranges will be made available on the GitHub repository, with date-based versioning. As more raw MI data becomes available through research, more dimensions such as construction periods can be easily added to our procedure. Likewise, with more and better data on other materials (e.g. architectural finishings), the same process demonstrated here can be used to expand to MI ranges beyond the eight main structural materials. In this regard, our estimation approach also highlights the inequality of current data coverage in different global regions, especially in the Global South, and the need for efforts by the scientific community to address these knowledge gaps. Furthermore, the code can be forked to differentiate MIs by other features than function type, structure type, or regions. Likewise, different definitions of features than the ones we use are possible, for example different region classifications than the 32 SSP regions, or breaking up highly aggregated regions with already rich data like the EU15 to sub-regions like North European EU countries and South European EU countries, thus capturing the material intensities of their unique construction styles.

## Data Availability

The entire workflow is available in the RASMI GitHub repository (https://github.com/TomerFishman/MaterialIntensityEstimator): the methods for creating the MI value ranges are implemented in Python 3 code, and the random forest classification of structure types is implemented in the Orange machine learning and data mining suite^58^. The GitHub repository also stores the resulting data and supporting figures. Released versions of the data are archived in Zenodo. The Zenodo version used for this data descriptor is v20230905^59^.
